# Pancreatic Schistosomiasis, China, 2020–2024

**DOI:** 10.3201/eid3112.251098

**Published:** 2025-12

**Authors:** Long He, Chuanbing Zhao, Yu Lu, Hongzhen Wei, Zanglong Deng, Yunpeng Zhang, Tao Yin

**Affiliations:** Union Hospital Department of Pancreatic Surgery, Tongji Medical College, Huazhong University of Science and Technology, Wuhan, China

**Keywords:** Pancreatic schistosomiasis, *Schistosoma*, schistosomiasis, parasites, zoonoses, parasitic infections, China

## Abstract

Schistosomiasis is a globally prevalent parasitic infection, but pancreatic involvement is extremely rare. In this article, we report 4 cases of pancreatic schistosomiasis from endemic regions in China. The possible link between schistosomiasis and pancreatic malignancy deserves further study. These cases underscore the diagnostic challenge of pancreatic schistosomiasis.

Schistosomiasis is a parasitic infection caused by trematodes of the genus *Schistosoma* (*1*). Globally, ≈1 billion people are at risk for schistosomiasis and there have been 250 million cases across 78 countries. By 2021, the disease accounted for >1.8 million disability-adjusted life years lost. *Schistosoma* infection is closely linked to impoverished living conditions (*2*).

The *Schistosoma* lifecycle requires an intermediate host, such as *Biomphalaria*, *Bulinus*, and *Oncomelania* snails, before infecting and causing disease in definitive hosts such as humans or other mammals (*3*). The main pathogenic species in humans are *S. hematobium*, *S. japonicum*, and *S. mansoni*. Although adult worms are relatively nonpathogenic, their eggs induce granulomatous inflammation characterized by eosinophilic and lymphocytic infiltration, leading to both acute and chronic disease (*4*).

The clinical manifestations of schistosomiasis are related to infection intensity and duration, and the intestines, liver, and bladder are the most involved organs (*2*). Early infection might manifest as cercarial dermatitis. Acute schistosomiasis is often asymptomatic, but sudden onset of fever, myalgia, fatigue, and abdominal pain lasting 2–10 weeks can occur. Chronic schistosomiasis is typically characterized by nonspecific, intermittent abdominal pain, diarrhea, and rectal bleeding (in *S. mansoni* and *S. japonicum* infections) or hematuria (in *S. hematobium* infection). In advanced chronic cases, complications such as portal hypertension (*S. mansoni* and *S. japonicum*) or hydronephrosis, renal failure, and bladder cancer (*S. hematobium*) can occur (*2*).

Emerging evidence suggests that schistosomiasis is a systemic disease that might also involve the spleen, heart, lungs, nervous system, and other organs (*5*–*7*). However, pancreatic involvement is exceedingly rare. In this article, we report 4 cases of schistosomiasis in China affecting the pancreas (Table). Informed consent was obtained from the patients for the publication of their information.

**Table:** Clinical characteristics of 4 patients with pancreatic schistosomiasis, China, 2020–2024

Case no.	Age, y/sex	Region of infection	Hemaglobin, g/L	*Schistoma japonicum* eggs detected	Stool test	Treatment for *S. japonicum*
1	49/F	Pancreas head	93	Yes	Positive	Praziquantel, 60 mg/kg, 3 doses in 1 day
2	73/M	Pancreatic body	94	Yes	Positive	Praziquantel, 60 mg/kg, 3 doses in 1 day
3	65/M	Pancreatic tail	125	Yes	Positive	NA
4	47/M	Pancreatic tail and spleen	106	Yes	Not available	Praziquantel, 60 mg/kg, 3 doses in 1 day

## The Study

**Figure 1:**
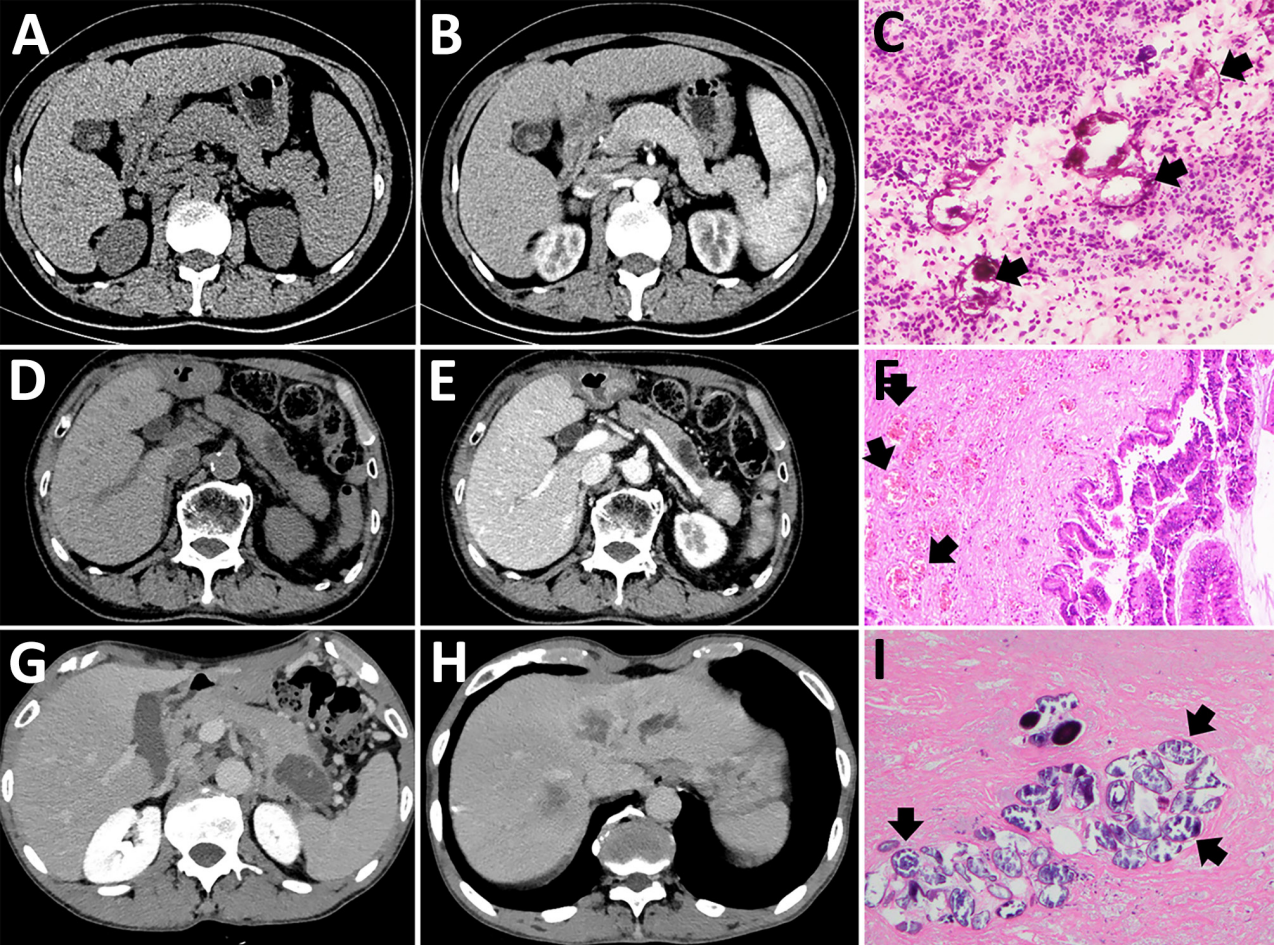
Imaging from 4 cases of pancreatic schistosomiasis, China, 2020–2024. A, B) Computed tomography (CT) images from patient 1 revealing a mass involving the pancreas. C) Pathologic examination from fine-needle aspiration of patient 1 showing fibrinous exudate and schistosome eggs (arrows). D, E) Abdominal CT of patient 2 showing a nonenhancing, tubular lesion adjacent to the main pancreatic duct in the body of the pancreas. F) Pathologic examination of sample from patient 2 showing intraductal papillary mucinous neoplasm with moderate dysplasia and scattered schistosome eggs (arrows). G, H) Abdominal CT of patient 3 revealing a low-density mass in the pancreatic tail and multiple ring-enhancing hepatic nodules. I) Pathologic examination of sample from patient 3 showing schistosome egg deposition (arrows) with associated tissue necrosis.

Patient 1 was a 49-year-old woman with a 1-year history of intermittent fever (up to 40°C) who was found to have a space-occupying lesion in the pancreatic head during routine examination in October 2020. She resided in Hubei, China, and had a history of untreated schistosomiasis. Computed tomography at Wuhan Union hospital revealed a 4.8 × 3.5 cm mass superior to the pancreatic head, involving the pancreas and associated with enlarged peripancreatic lymph nodes (Figure 1, panels A, B). Tumor markers were unremarkable, but hemoglobin was mildly reduced (93 g/L, reference range 115–150 g/L). To clarify the diagnosis, we conducted an endoscopic ultrasound-guided fine-needle aspiration of the pancreatic head. Pathology demonstrated fibrinous exudate and schistosome eggs (Figure 1, panel C). *S. japonicum* eggs were also found in the stool. The patient was diagnosed with pancreatic schistosomiasis and treated with a 1-day course of praziquantel (60 mg/kg in 3 doses). Two weeks later, her symptoms resolved, and she was discharged. No fever recurrence was observed at 3-month follow-up.

**Figure 2:**
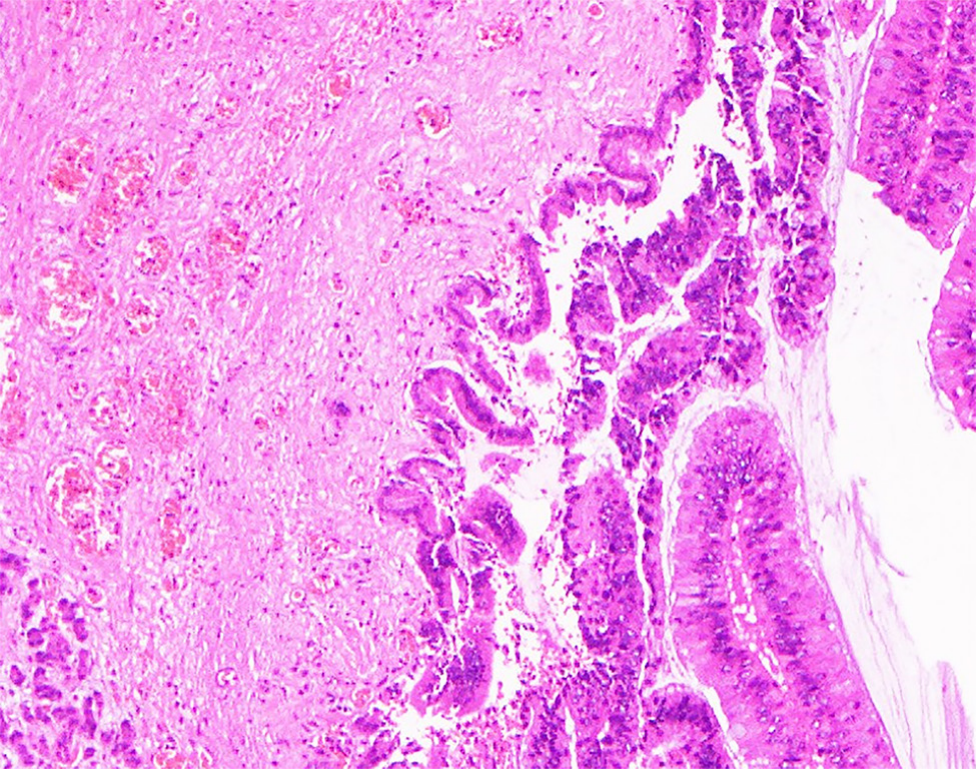
Enlarged image of schistosome eggs in pancreas of patient 2 in report of 4 cases of pancreatic schistosomiasis, China, 2020–2024.

Patient 2 was a 73-year-old man who sought care in November 2024 with 1 day of abdominal pain. Six months prior, he had a history of pancreatitis. He also had a long-term residence in Hubei and a history of schistosomiasis without any treatment. Laboratory findings included hemoglobin 94 g/L (reference range 130–175 g/L) and serum amylase 522 U/L (reference range 20–125 U/L); tumor markers were unremarkable. *S. japonicum* eggs were detected in his stool. Abdominal computed tomography scan revealed a nonenhancing, tubular lesion adjacent to the main pancreatic duct in the body of the pancreas, suggesting possible intraductal papillary mucinous neoplasm (IPMN) and an enhancing nodule in the pancreatic tail, suspected to be a neuroendocrine tumor (Figure 1, panels D, E). After fluid replacement, analgesia, and resumption of oral intake, the abdominal pain resolved. We conducted a laparoscopic distal pancreatectomy. Pathology revealed IPMN with moderate dysplasia in parts of the glandular epithelium, and scattered schistosome eggs (Figure 1, panel F; Figure 2). We discharged the patient 2 weeks postoperatively and referred him to the infectious diseases department. He was treated with a 1-day course of praziquantel (60 mg/kg in 3 doses). At 3-month follow-up, he remained symptom-free.

Patient 3 was a 65-year-old man who sought care in March 2022 with complaints of left upper abdominal pain lasting 1 month. He had a history of liver cirrhosis and untreated schistosomiasis. Hepatitis B surface antigen, e antigen, and core antibody were all positive. Tumor markers carcino-embryonic antigen, CA125, and CA19-9 were markedly elevated. Hemoglobin measured slightly low at 125 g/L (reference range 130–175 g/L). Contrast-enhanced abdominal computed tomography revealed a low-density mass in the pancreatic tail, highly suggestive of pancreatic cancer, and multiple ring-enhancing hepatic nodules suggestive of metastases (Figure 1, panels G, H). Endoscopic ultrasound-guided biopsy yielded 2 tissue samples: 1 sample showed schistosome egg deposition with tissue necrosis, and the other sample revealed pancreatic carcinoma cells (Figure 1, panel I). We diagnosed the patient with pancreatic schistosomiasis and pancreatic ductal adenocarcinoma with liver metastases. We transferred him to the oncology department for further management. Four months later, follow-up revealed the patient had died.

We published the case of patient 4 last year (*8*). In brief, a 47-year-old man who had had intermittent left upper quadrant abdominal pain for 5 years was found to have a hypodense cystic lesion in the tail of the pancreas on imaging. He subsequently underwent surgery, and the resected specimen’s pathology confirmed pancreatic and splenic schistosomiasis. Splenic schistosomiasis was documented previously (*9*). 

## Conclusions

During the past 70 years, China has made remarkable progress in controlling schistosomiasis (caused by *S. japonicum*), resulting in a 99% reduction in prevalence. China is steadily progressing toward the goal of complete schistosomiasis elimination (*10*). However, sporadic cases of schistosomal infection continue to occur in endemic areas such as Hunan, Jiangxi, and Hubei.

Pancreatic schistosomiasis is extremely rare, and we found only 1 known previous report of chronic pancreatitis caused by *S*. *mansoni* (*11*). Animal studies have shown that schistosome eggs can be deposited in pancreatic tissue (*12*). All 4 patients described in this article lived in endemic areas and had a history of schistosomiasis. Their main symptoms were intermittent abdominal pain and fever, and imaging consistently indicated pancreatic space-occupying lesions that posed diagnostic challenges. Two cases were ultimately diagnosed as inflammatory lesions, 1 as IPMN, and 1 as pancreatic ductal adenocarcinoma with hepatic metastases. Pathology confirmed *S*. *japonicum* egg deposition in all 4 cases.

Egg deposition in the pancreas induces granuloma formation and chronic inflammation. Of note, in case 3, pancreatic cancer cells and liver metastasis were found at the lesion site. Associations between schistosomiasis and colorectal, liver, and bladder cancers have been previously published (*13*–*15*). Furthermore, schistosome egg deposition in colorectal cancer is linked to a higher rate of the KRAS G12D mutation, which is also a key driver mutation in pancreatic cancer. Therefore, the pancreatic cancer in case 3 might be related to schistosomal infection, rather than being coincidental.

Once pancreatic schistosomiasis is confirmed, praziquantel remains the primary treatment. In this article, patient 1 improved with praziquantel therapy; patients 2 and 4 underwent surgery because of the high suspicion of malignancy and were treated with praziquantel after the diagnosis of pancreatic schistosomiasis; and patient 3 primarily received chemotherapy but died because of advanced disease.

Schistosomiasis involving the pancreas is exceedingly rare and manifests with nonspecific symptoms, making diagnosis challenging. In endemic areas, particularly in patients with a history of schistosomiasis who are found to have pancreatic lesions, pancreatic schistosomiasis should be considered as part of a differential diagnosis. In addition, there is evidence of a potential association between *Schistosoma* infection and pancreatic cancer.
